# Evaluation of *δ*^15^N analysis to trace the origin of *Diaphorina citri* (Hemiptera: Liviidae) to citrus orchard fertilization management

**DOI:** 10.7717/peerj.8807

**Published:** 2020-03-27

**Authors:** Francesc Gomez-Marco, Andrew Yoshimoto, W. Evan Braswell, Richard Stouthamer

**Affiliations:** 1 Department of Entomology, University of California Riverside, Riverside, CA, United States of America; 2 PPQ, Mission Laboratory, Edinburg, TX, United States of America

**Keywords:** Organic management, Asian citrus psyllid, Conventional management, Stable isotopes

## Abstract

We investigated the variability of nitrogen stable isotope ratios ^15^N/^14^N (expressed as δ^15^N) on citrus orchards with different fertilization management practices (organic versus conventional) and its correlation with the δ^15^N values of the key citrus pest *Diaphorina citri* Kuwayama (Hemiptera: Liviidae) feeding on such plant material. Tracing the origin of this pest in open field is crucial since the insect is a vector of the incurable and devastating citrus disease known as Huanglongbing. We hypothesized that the origin (natal tree) of the pest may be deduced by correlating the δ^15^N values obtained from the young citrus leaves and from adults of *D. citri* raised on them. First, laboratory experiments were performed to understand the acquisition and incorportation of the δ^15^N values by *D. citri*. Second, we confirmed the positive correlation between the δ^15^N values of the young citrus leaves and *D. citri*. Finally, field sampling was carried out in 21 citrus orchards from Southern California to study the variability on the δ^15^N values on organic and conventional commercial citrus orchards. Laboratory results suggest that the analyses of the δ^15^N values can be regarded as a useful method to trace the origin of the pest. However, the high variability in nitrogen resource used in both fertilization management practices (especially in organic orchards) by growers makes the application of this technique unfeasible to pinpoint the origin of *D. citri* in the citrus agroecosystem.

## Introduction

Stable isotopes of an element share the same atomic number but differ in mass due to variations in the number of neutrons. These elements, called poly-isotopics, have multiple stable forms that coexist in the environment reaching variable stages of equilibrium (Aston, 1921; Thomson, 1913). The equilibrium ratios can be vertically transferred along the food chain through consumption (Kelly, 2000; McCutchan Jr et al., 2003). Variation of stable isotope ratios through trophic levels have proven useful in traceability studies aimed at food quality control (Bateman, Kelly & Jickells, 2005; Camin et al., 2011; Rogers, 2008; Rossmann, 2001), organismal (Heinrich & Collins, 2017; Hobson, Wassenaar & Taylor, 1999; Ouyang et al., 2014) and system ecology (Boecklen et al., 2011; Boyer, Snyder & Wratten, 2016; Gratton & Denno, 2006; Hood-Nowotny & Knols, 2007; Hyodo et al., 2010; Scheunemann et al., 2016). Additionally, fertilizers or diets that differ in their isotopic composition can be used to study their effects on plant and animal isotope abundance among individuals of the same species (Bateman, Kelly & Jickells, 2005; DeNiro & Epstein, 1978; Oelbermann & Scheu, 2002; Tillberg & Breed, 2004; Webb, Simpson & Hedges, 1998).

Nitrogen (N) is one of the bio-elements of the ecosystem that is poly-isotopic and has been widely used in agriculture, food science (Camin et al., 2011; Mariotti et al., 1981) and ecology studies (Boecklen et al., 2011; Boyer, Snyder & Wratten, 2016; Gratton & Denno, 2006; Hood-Nowotny & Knols, 2007; Hyodo et al., 2010; McCutchan Jr et al., 2003; Ouyang et al., 2014; Scheunemann et al., 2016). Previous works have made relevant progresses in quantifying N isotope patterns of plants and soils at local to global scales as well as the mechanisms that underlie these patterns including the possible difficulties that come with interpreting isotope data (e.g., species-specific fractionation rates) (Craine et al., 2015). In general, interpretation of the nitrogen stable isotope ratio ^15^N/^14^N (expressed as δ^15^N) values relies on the premise that they are determined by the diet of consumers (McCutchan Jr et al., 2003). Deamination and transamination enzymes in animals prefer ^14^N, resulting in whole animal δ^15^N that is approximately 3‰ higher than its diet. Hence δ^15^N is also useful as an indicator of trophic level (McCutchan Jr et al., 2003; Minagawa & Wada, 1984).

The δ^15^N in plant material is strongly influenced by the primary source of nitrogen (Evans, 2001), and has been shown to be one of the most useful indices used to distinguish between organically and conventionally cultivated crops, including citrus (Bateman, Kelly & Jickells, 2005; Camin et al., 2011; Rapisarda et al., 2005). Synthetic fertilizers derived from atmospheric nitrogen (δ^15^N_atm_ = 0‰) in the Haber process have low δ^15^N and are not permitted for organic production (Bateman & Kelly, 2007; CDFA, 2019; USDA, 2019). Instead, soil fertility for organic agriculture relies on selected fertilizers which may be permitted by an inspecting authority (i.e., USDA Organic guidelines). These include products of plant and animal origin such as animal manures, composts, rape seed meal, blood meal and fish meal ( CDFA, 2019; USDA, 2019).

The citrus industry in California has an economic impact of about $7 billion per year, and California is the state with the largest share of organic production, with around 4.1 million of hectares in 2016 (California Department of Food and Agriculture , 2019; USDA, 2019). The California citrus industry has been threatened since 2008 by the arrival of the Asian citrus psyllid, *Diaphorina citri* Kuwayama (Hemiptera: Liviidae) (Grafton-Cardwell, Stelinski & Stansly, 2013), vector of the bacterium *Candidatus* Liberibacter asiaticus (CLas) (Bové, 2006). CLas is transmitted into citrus plants through the psyllid feeding and it causes the incurable and devastating disease known as citrus greening or Huanglongbing (HLB) (Bové, 2006). HLB-infected trees show symptoms of leaf and flush yellowing and they bear few, deformed, small and un-flavorful fruits (Bové, 2006). The life cycle of *D. citri* includes five nymphal stages before the winged adult stage, which can disperse and infest new trees (Grafton-Cardwell, Stelinski & Stansly, 2013). Two factors have proved important for the spread of HLB by its vectors. First, the nymphal stages of *D. citri* have low mobility and they stay within one branch of a single tree. However, nymphs readily acquire CLas if they feed on infected parts of the tree and transmit, as adults, to healthy trees. Second, adults grown on a healthy tree have low chances to become HLB vectors if they feed on HLB infected tree sporadically (Inoue et al., 2009; Pelz-Stelinski et al., 2010). For these reasons, it should be possible to locate the citrus trees (or orchards) that are HLB infected (HLB reservoirs) if we are able to track the origin of HLB-positive *D. citri* adults captured.

Different methods have been developed to mark and recapture *D. citri* (Boina et al., 2009; Nakata, 2008). However, biogeochemical markers, such as stable isotope ratios, do not require marking and recapturing individuals, and they provide time-integrated information that can be obtained passively to monitor seasonal migration of the populations (Peterson & Fry, 1987; Rubenstein & Hobson, 2004). In an experiment conducted to determine long-range dispersal capacity in the absence of severe weather events, *D. citri* were able to disperse at least 2 km within 12 days (Lewis-Rosenblum et al., 2015). Nevertheless, other studies suggests that adult *D. citri* barely moved once they had colonized a host plant (Kobori et al., 2011). Therefore, differentiating between *D. citri* adults coming from organic and conventional orchards by δ^15^N values would be of immediate practical benefit in making appropriate strategic decisions on Integrated Pest Management (IPM) citrus programs. In addition, inferring the origin of HLB-infected insects with δ^15^N values would facilitate narrowing the zones with HLB infected trees at a local scale. Such a method would have a low-cost (around $6 per specimen) in the actual cost of the techniques used to monitor the pest movement and it could better inform where they are feeding in a specific season to apply prospective treatment options against the pest. Finally, this technique may improve food quality control studies, since being completely able to differentiate between organic and conventional crops may have a high impact on market prices and competitivity of the citrus industry (Camin et al., 2011; Rapisarda et al., 2005; Rogers, 2008).

In this study we hypothesize that i) δ^15^N values acquired during the nymphal stages of *D. citri* will be conserved in the final developmental stage (adult), ii) δ^15^N values from young citrus leaves will be highly correlated with the δ^15^N values from the pest, *D. citri* and iii) citrus orchard fertilization management can be traced using δ^15^N values of young leaves. If the three hypotheses are correct, we may be able to trace the citrus orchard (or area) of origin of individual specimens of *D. citri.* To test the first hypothesis, we conducted a laboratory experiment with *D. citri* using different fertilization treatments on the citrus relative host plant *Murraya koenigii* (L.) Sprengel (Sapindales: Rutaceae), which is commonly used for mass rearing of this insect. To test the second hypothesis, we performed a field experiment in four citrus orchards to correlate the δ^15^N values of the *D. citri* and the δ^15^N values of the plant material where the insects were feeding. Lastly, to test the third hypothesis we sampled leaves from a large number of commercial citrus orchards from Southern California labeled as organic or conventional production (Table 1).

**Table 1:** Citrus orchards information. Dates in bold indicates that the orchads were sampled in two sampling dates and “-” indicates unknown information.

**Management**	**Variety**	**Orchard number**	**Sampling dates**	**Nitrogen resource**	**Fertilizer (dosage) [N-P-K]**	**Size (ha)**	**Location**
Conventional	Navel	C1	**Mar-17/Jul-17**	Urea, Ca(NO_3_)_2_	403.51 kg/ha of Ca(NO_3_)_2_ spread. Foliar treatments of 56.04 kg/ha of urea in August and 11.21 kg/ha in November	3.642	Redlands
		C2	Mar-17	Urea, Ca(NO_3_)_2_	403.51 kg/ha of Ca(NO_3_)_2_ spread. Foliar treatments of 56.04 kg/ha of urea in August and 11.21 kg/ha in November	4.047	Redlands
		C3	**Dec-16/Mar-17**	UN-32	(15-5-5)	4.261	Riverside
		C4	**Dec-16/Mar-17**	UN-32	(15-5-5)	8.176	Riverside
		C5	**Mar-17/Oct-17**	UN-32	–	1.94	Redlands
	Valencia	C6	Mar-17	Urea, KNO_3_	22,42 kg/ha of urea and 22,42 kg/ha of KNO_3_ in May	19.425	Loma Linda
		C7	Mar-17	Ca(NO_3_)_2_	403.51 kg/ha of Ca(NO_3_)_2_ spread. 22.42 kg/ha of urea and 22.42 kg/ha of KNO_3_ in May	3.642	Redlands
		C8	Mar-17	Urea, Ca(NO_3_) _2,_KNO_3_	403.51 kg/ha of Ca(NO_3_)_2_ spread. 22.42 kg/ha of urea and 22.42 kg/ha of KNO_3_ in May	6.070	Loma Linda
		C9	May-18	–	–	1.509	Lindsay
		C10	May-18	–	–	3.365	Lindsay
Organic	Navel	O1	**Mar-17/Jul-17**	Manure	Chicken pellets once a year	0.809	San Diego
		O2	Mar-17	–	True (10-2-8) blended organic fertilizer in July, Oct and March	0.809	Temecula
		O3	**Dec-16/Mar-17**	Urea	Urea (56.04 kg/ha), Phosphorous acid (2.24 kg/ha), Zinc Manganese (5.6 kg/ha)	8.357	Riverside
		O4	**Mar-17/Oct-17**	Urea, manure, feather meal	(4-4-2)/(16-0-0)	0.887	Redlands
	Valencia	O5	May-17	Vegetable matter, manure and aquaculture residuals	Pyganic (MGK), Oro Boost, Entrust SC, Compost (American Organics), Earthworm Organics Vermigrow tea	1.214	Salton Sea
		O6		Urea, soy protein hydrolase, seaweed extract, (NH_4_)_2_SO_4_, NH_4_NO_3_, (NH_4_)_2_HPO_4_, KNO_3_	Explorer, Nutra Plus, Acadian seaweed, OMG, N-Texx, Ferticel (5-10-10)	6.741	Lindsay
		O7				3.977	Lindsay
		O8	May-18			3.963	Lindsay
		O9				4.053	Lindsay
		O10				5.863	Lindsay
		O11				7.206	Strathmore

## Material and Methods

### Impact of developmental stage on *Diaphorina citriδ*^15^N values incorporation

*Diaphorina citri* adults 4 to 7 days old (age of *D. citri* adults referred as days after emergence) were obtained from a colony established at the University of California Riverside (UCR). This colony has been reared on *M. koenigii* plants since 2011 in environmental rooms (∼27 °C, ∼50% RH, 8:16 D:L, light source; Sylvania Octron 4100k fo32/741 32 w) in the Insectary and Quarantine Facility at UCR. Collected *D. citri* adults were sorted with a 1:1 sex ratio in a mating cage that contained *M. koenigii* seedlings on an environmental room with the same conditions specified above. These seedlings were sown and grown in approximately 1g mixture of Vermiculite and Pearlite (2:1) without fertilization treatment inside of an 18 × 150 mm borosilicate glass culture tube (Fisher Scientific, Hampton, NH, USA). Seedlings developed until their stem reached approximately 8 cm in height with more than 5 leaves. 100 *D. citri* adults 9 to 11 days-old were confined inside the cages for mating and oviposition for four days. After the confinement period, the adults were removed, and their offspring developed until adult stage on the seedlings. The resulting *D. citri* adults were used as the initial population (F_0_) for the experiment. Seeds of *M. koenigii* were collected from two trees of the UCR Citrus Variety Collection. Upon collection, seeds were extracted from the fruit and washed to remove most of the extraneous flesh before being sterilized in a 2% NaClO solution. Thereafter, 300 seeds were split evenly into two groups in a greenhouse: the first group labeled as “organic” being fertilized with an organic OMRI certified bone meal fertilizer (Jobe’s Organics, 2-14-0 NPK), while the other group labeled as “conventional” was treated with Osmocote (14-14-14 NPK). The assigned fertilizer was applied (52 mg/pot) to the seeds upon sown and every three months until the *M. koenigii* plants were 6 to 8 months old.

The *F*_0_ *D. citri* adults were divided equally between organic and conventional treatments and were exposed to six *M. koenigii* plants (initial plants) from each fertilization treatment for five days (in environmental rooms at ∼27 °C; ∼50% RH, 8:16 D:L, light source; Sylvania Octron 4100k fo32/741 32 w). After oviposition, the insects were removed and stored at −20 °C. The resultant offspring growing on each plant were removed from their initial plant/treatment at different stages of development (2nd instar nymphs, 4th instar nymphs and adults 0 to 1, 0 to 3 and 0 to 7 days old [∼20 *D. citri* nymphs or adults on each of the three plants assigned to a specific “*D. citri* stage/age transferred” namely as “group of psyllids” and treatment for a total of *n* = 30 plants]) and randomly transferred to a new *M. koenigii* plant with the opposite fertilization treatment (the individuals that hatch on the plants with organic treatment were transferred to plants with conventional treatment and the opposite) (Fig. S1). After being transferred to their new host plat/treatment, all *D. citri* individuals from the 30 *M. koenigii* plants developed to adulthood and continued to feed for 12 days after emergence on the last plant/treatment. On day 12, they were removed from the plant and stored at −20  °C. From each plant, two to six adults were selected to perform δ^15^N analyses (see ‘Plan material and *D. citri* sample δ ^15^N analysis). Three leaves per plant for a total of 42 plants (12 plants where the F_0_ oviposited and 30 plants to which the insects were transferred) were processed to analyze the δ^15^N (see Plant material and *D. citri* sample δ ^15^ N analysis).

### Relationship between the citrus leaf and *D. citriδ*^15^N values

Four citrus orchards (C1, C4, O1 and O4) (Table 1) were surveyed for the presence of *D. citri* populations, during the summer of 2017 (May-August), when *D. citri* populations usually peak in Southern California (Milosavljević et al., 2018). Ten to 20 trees in each grove with at least two *D. citri* colonies per tree were selected. White paint filter bags of 3.8 L (Workforce, China) were used as exclusion bags and secured with a zip-tie over each infested flush to isolate *D. citri* colonies with a maximum of three colonies per tree. Exclusion bags were checked weekly until more than 50% of the nymphs were either deceased or emerged as adults (71 exclusion bags with adults obtained), then the full branch was trimmed and stored at −20  °C until δ^15^N analyses of plant and insect material (see ‘Plant material and insect sample δ^15^N analysis’).

### Field sampling for citrus leaf δ^15^N analysis

A total of 21 citrus orchards were sampled in the South and Central regions of California for δ^15^N analysis, consisting of 12 Valencia and 9 Navel sweet orange groves (*Citrus sinensis* (L.) Osbeck) all with Carrizo citrange as the rootstock (Table 1). Out of these 21 orchards, 11 were managed according to USDA organic guidelines and 10 were managed according to conventional methods (not organic) (Table 1). At each grove, a transect was selected to best encompass the presence of *D. citri* (checked by visual inspection) and the range in soil and environmental differences (a diagonal transect of the grove selecting a tree every other row when the *D. citri* presence allow it). Ten to 18 trees that fell upon this transect were randomly selected, and five young leaves (leaves from the most recent flushing period, light-dark green and fully expanded leaves) on different branches were collected at eye level (1.5 to 2 m above soil level) and stored at −20 °C until processed (see Plant material and *D. citri* sample δ^15^N analysis). Of these, we selected five trees randomly and three leaves for each were analyzed to compare leaves values of δ^15^N between conventional and organic orchards. Values of δ^15^N obtained from the plant material in the same citrus orchards but in different experiments from this study were also included in this analysis (see Relationship of the citrus leaf and *D. citri* δ^15^N values).

Six orchards (C1, C3, C4, O1, O3 and O4) were sampled on different seasons to assess seasonal variation of δ^15^N values of citrus leaves (Table 1). The six orchards were surveyed on March of 2017 (Spring). Additionally, the orchards C3, C4 and O3 were surveyed in December of 2016 (Winter) and C1, O1 and O4 were surveyed in July of 2017 (Summer).

Field experiments of this and the previous section were approved by the United States Department of Agriculture’s Animal and Plant Health Inspection Service (APHIS) (project number: #16-8130-0668-CA).

### Plant material and *D. citri* sample *δ*^15^N analysis

Citrus and *M. koenigii* leaves were rinsed with deionized water three times before being dried at 60 °C in an Isotemp hybridization incubator (Fisher Scientific, Hampton, NH, USA) for 48 h. *D. citri* individuals were dried at 55  °C for 24 h or constant dry weight in the same incubator as the plant material. Leaves were individually ground into a fine powder using a mortar and pestle. *D. citri* samples were not ground and all material was stored separately in 2 mL microtubes. Target sample mass was calculated according to University of California, Davis Stable Isotope Facility (SIF) standards. Each sample was weighted in a 0.0001 g resolution scale (Intelligent Weighing Technology, Camarillo, CA, USA) then enclosed in 9 × 5 mm tin capsules (Elemental microanalysis, Okehampton, UK) and sealed to prevent leakage or contamination during the shipment. Samples were shipped to the University of California, Davis SIF in 96 well plates and analyzed using a PDZ Europa ANCA-GSL elemental analyzer interfaced to a PDZ Europa 20–20 isotope ratio mass spectrometer (Sercon Ltd., Cheshire, UK).

### Statistics

All the statistical analyses described below were done in R (http://www.R-project.org). The difference between fertilization management on the δ^15^N values of the plant material in the experiment of δ^15^N values incorporation on the different *D. citri* stages were compared by one way ANOVA followed by Bonferroni’s posthoc tests for multiple comparison for all group of psyllids. The δ^15^N values for the insects in the same experiment were not independent since the source of all the insects for each treatment was from the same group of plants. Therefore, these values were analyzed using a linear mixed effects model with developmental stage (group of psyllids) as random factor with the “R” packages “*car*” and “*nlme*”. After this we ran pairwise comparisons between fertilizations for each group of psyllids using estimated marginal means with Tukey’s adjustment with the “R” package “*emmmeans*”. The average δ^15^N values of the citrus leaves and *D. citri* individuals from each exclusion cage installed on the citrus canopies were related with a linear regression. The citrus leaf δ^15^N values between seasons within the orchard were analyzed by Student’s *t*-test (see Citrus leaf δ ^15^N values on different seasons result). Finally, the δ^15^N values by leaf of the 21 citrus orchards sampled to assess differences between fertilization managements were analyzed by ANOVA, and the average of the citrus leaf δ^15^N values by orchard data were modeled using a kernel density estimation for the distribution of between-group (conventional and organic) variability with the “R” packages *ggplot2*, *plyr* and *sm*.

## Results

### Impact of developmental stage on *Diaphorina citriδ*^15^N values incorporation

**Figure 1:**
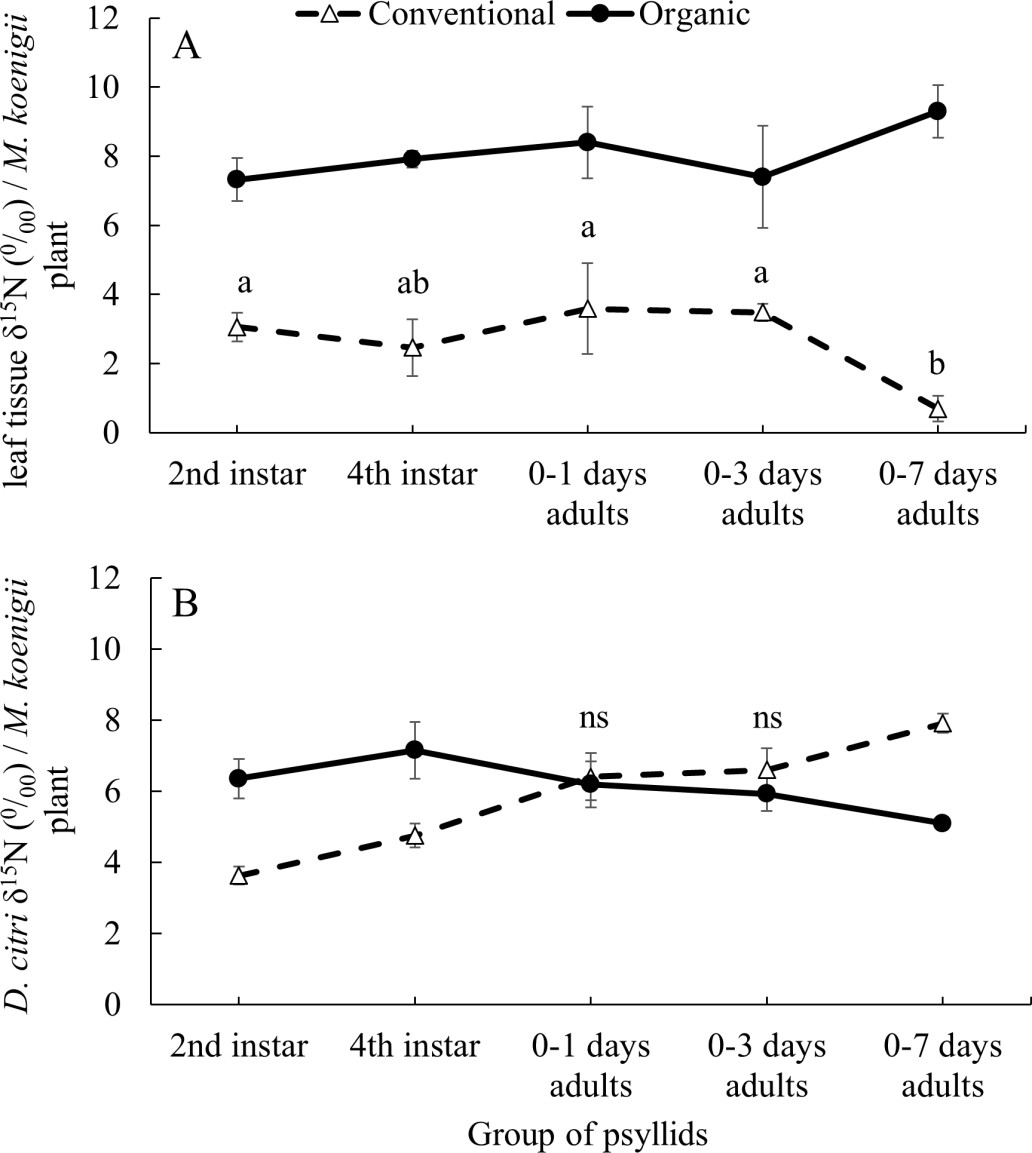
Impact of developmental stage on *D. citriδ*^15^N values incorporation. Average *δ*^15^N values (±SE) for (A) leaf tissue from *M. koenigii* plants (with two fertilization treatments: conventional and organic) used to feed *D. citri* transferred from the opposite fertilization treatment (different letters indicate significant differences between group of psyllids (transference time) on the same fertilization treatment) (B) *D. citri* adults transferred from the opposite fertilization treatment plants on different stages (2nd instar: when the *D. citri* nymphs were 2nd instar nymphs and successively). Black circles indicate those *D. citri* raised on conventional and transferred to organic on that specific transference time and white triangles the opposite (“ns” indicates no significant differences between fertilization treatments of the same group of psyllids). Note that all the adult individuals from each groups of psyllids were analyzed for its *δ*^15^N values at 12 days after their emergence.

The average δ^15^N values of the 41 *M. koenigii* plants grown with organic (8.139 ± 0.199‰) and conventional (2.867 ± 0.197‰) fertilization treatments were significantly different (*F*_1,124_ = 355.23, *P* < 0.001) (Fig. 1A, note that the initial *M. koenigii* plants, where the F_0_ oviposit, for each treatment are included in the analysis but not in the figure). The plants from the organic treatment assigned to each group of psyllids did not differ significantly on its δ^15^N values (*F*_5,57_ = 2.166, *P* = 0.071). However, the δ^15^N values from the plants with conventional treatment assigned to each group of psyllids differed significantly (*F*_5,57_ = 6.989, *P*  < 0.001) (Fig. 1A).

The δ^15^N values from the F_0_ *D. citri* adult population (those psyllids raised on non-fertilized plants and that laid the eggs on the fertilized initial *M. koenigii* plants) did not significantly differ after being fed, as adults, on conventionally or organically fertilized *M. koenigii* plants for five days (*F*_1,18_ = 2.659, *P* = 0.12). However, their offspring, which spent some time feeding on fertilized plants as nymphs, had higher δ^15^N values (F_0_ *D. citri* adults δ^15^N = 0.945 ± 0.307‰; *n* = 20).

The values of δ^15^N on the *D. citri* adults obtained were not influenced by the fertilization treatment (*χ* ^2^ = 0.453, *df* = 1, *P* = 0.501) or the group of psyllids (*χ*^2^ = 2.794, *df* = 4, *P* = 0.593), but the interactions between these two variables was significant (*χ*^2^ = 123.384, *df* = 4, *P* < 0.001). The average δ^15^N values of *D.citri* adults raised on *M. koenigii* plants with conventional fertilization and transferred to plants with organic fertilization during the 2nd and 4th nymphal stages were higher (6.356 ± 0.551 and 7.154  ± 0.156‰  for 2nd and 4th respectively) compared to those same stage transferred from organically to conventionally managed plants (3.629 ±  0.247‰ and 4.752 ±  0.335‰ for 2nd and 4th respectively) (2nd: *P* < 0.001; 4th: *P* < 0.001) (Fig. 1B). However, *D. citri* adults that spent their entire nymphal period feeding on conventional or organic plants prior to being transferred to the opposite treatment 0–1 and 0–3 days after emerging as adults and feeding on their new hosts for 12 days, did not differ in the δ^15^N values between fertilization treatments (0–1 days: *P* = 1; 0–3 days: *P* = 0.844) (Fig. 1B). Conversely, *D. citri* adults transferred from organic plants to conventional plants at the stage “0–7 days old” had an average value of δ^15^N, 7.914 ± 0.274‰, higher than those transferred from conventional plants to organic plants at the same stage [5.098 ± 0.156‰, *P*  <  0.001] (Fig. 1B).

### Relationship between the citrus leaf and *D. citri*δ^15^N values

**Figure 2:**
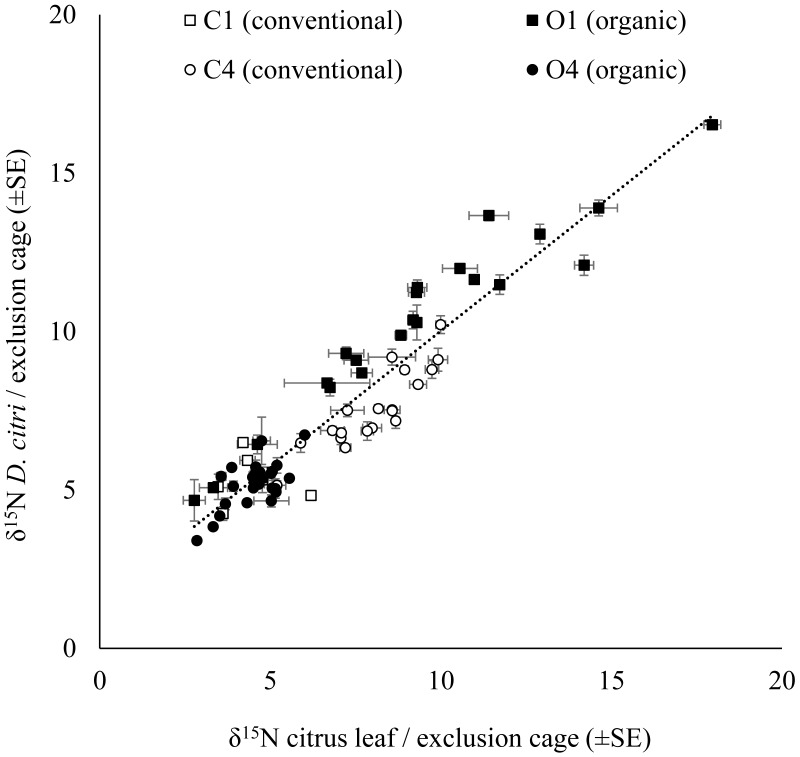
Relationship of the citrus leaf and *D. citriδ*^15^N values. Positive correlation of average *δ*^15^N values (±SE) on citrus young leaves and the average *δ*^15^N values (±SE) of the *D. citri* feeding on each exclusion cage from four citrus orchards (C1, C4, O1 and O4) with two different fertilization managements (white marker for conventional and black marker for organic). Linear regression (dotted line): (*δ*^15^N *D. citri*) = 0.854 * (*δ*^15^N citrus leaf) + 1.479, *R*^2^ = 0.88, *P* < 0.001.

The δ^15^N values for *D. citri* adults obtained in each exclusion cage were positively correlated with the δ^15^N values of the young citrus leaves from the same exclusion bag (Fig. 2). However, there were no significant influence of the fertilization managements (conventional vs. organic) on either the δ^15^N values of the leaves from the exclusion bags (avg. 6.774 ± 0.178‰, *n* = 271; *F*_1,67_ = 0.623, *P* = 0.433) or on the δ^15^N values of the *D. citri* adult individuals reared inside those exclusion bags (avg. 7.272 ± 0.169‰  *n* = 258; *F*_1,67_ = 1.379, *P* = 0.245). Therefore, it was not possible to trace the origin of the individuals. The difference between the average δ^15^N values of the leaves and the δ^15^N values of the *D. citri* adults per cage showed and an increase of 0.471 ± 0.125 (*n* = 71).

### Citrus leaf δ^15^N values in different seasons

**Figure 3:**
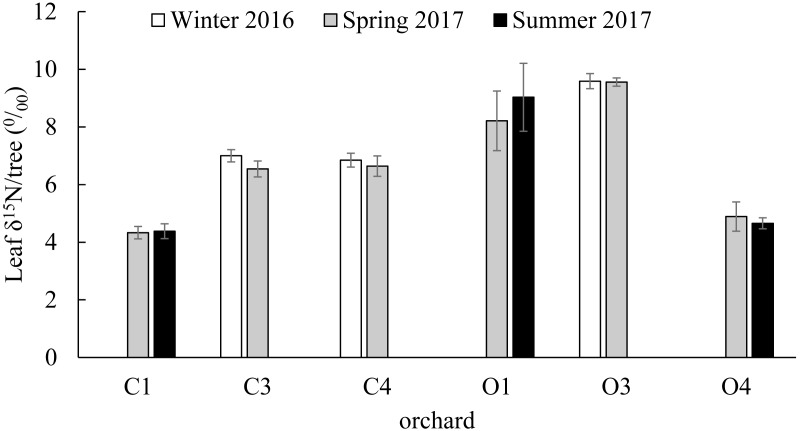
Citrus leaf *δ*^15^N values in different seasons. *δ*^15^N (‰) average values (±SE) of young citrus leaf tissue from six citrus orchards (C1, C3, C4, O1, O3 and O4) in three different seasons; Winter of 2016 (December), Spring of 2017 (March) and Summer of 2017 (July). C3, C4 and O3 sampled in December of 2016 and March of 2017 (all orchards and periods *n* = 5). C1, O1 and O4 sampled in March of 2017 (all orchards *n* = 5) and July of 2017 (C1 and O1 *n* = 9, O4 *n* = 17). No significant differences were found in each orchard between seasons.

No significant differences were found in the citrus leaf δ^15^N values between seasons within orchards (C1; *t*(36) = 0.91, *P* = 0.437, C3; *t*(28) = 1.39, *P* = 0.176, C4; *t*(21) = 0.63, *P* = 0.534, O1; *t*(23) = 1.36, *P* = 0.186, O3; *t*(21) = 0.08, *P* = 0.936, O4; *t*(16) = 0.9, *P* = 0.381). The δ^15^N values of the 239 young citrus leaves analyzed from six orchards (C1, C3, C4, O1, O3 and O4) varied between a minimum of 2.352 ‰ in O1 in Summer of 2017 and a maximum of 18.393‰ in the same orchard in Spring of 2017. From the citrus orchards compared in winter (December 2016) and spring (March 2017) (C3, C4 and O3), O3 had the highest average citrus leaf δ^15^N value with 9.589 ± 0.261‰ and 9.557 ± 0.143‰ in winter and spring respectively (Fig. 3). From the citrus orchards compared in spring (March 2017) and summer (July 2017) seasons (C1, O1 and O4), the highest average citrus leaf δ^15^N values were obtained from O1 with 8.212 ± 1.035‰ and 9.029 ± 1.179‰ in Spring and Summer respectively (Fig. 3).

### Citrus leaf δ^15^N values in conventional and organic groves

**Figure 4:**
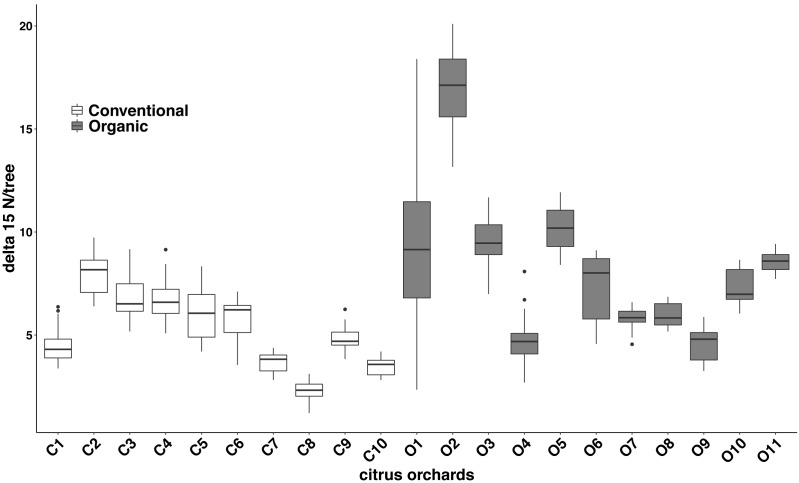
Citrus leaf *δ*^15^N values in conventional and organic groves. Average *δ*^15^N (‰) values (±SE) of 21 citrus orchards under different fertilization managements (Table 1) (dark grey for organic orchards: *n* = 11; white for conventional: *n* = 10; black points indicate the outliers).

**Figure 5:**
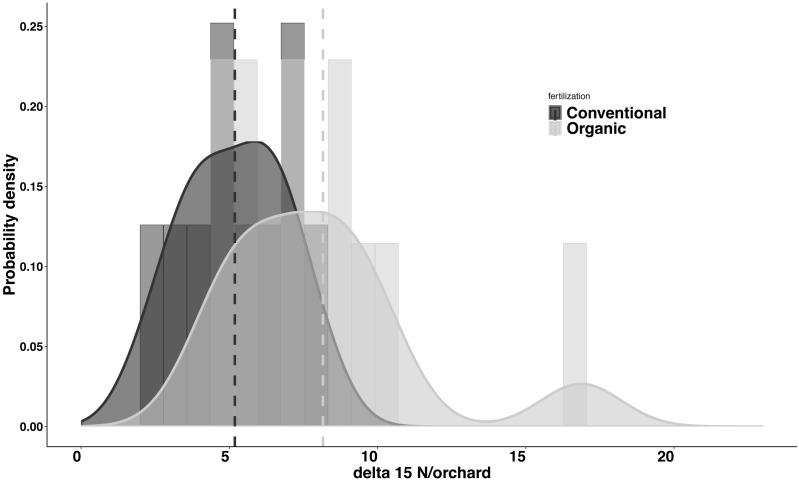
Probability density for average *δ*^15^N values for citrus orchards. Modeled kernel density estimation curves and histogram of the raw data for the organic (light grey; *n* = 11) and conventional (black curve and dark grey; *n* = 10) average citrus leaf *δ*^15^N values per orchard. The dashed lines show the average for each data group.

Significant differences were found in the δ^15^N values between fertilization managements (*F*_1,548_ = 79.244, *P* < 0.001). On average, the organic and the conventional orchards had a δ^15^N value of 8.158  ± 1.038‰ and 5.185 ± 0.561‰ respectively. The maximum δ^15^N value (20.103‰) was detected in an organic orchard (O2) and the minimum δ^15^N value (1.215‰) was obtained from a conventional orchard (C8) (Fig. 4). Modeled Kernel distribution density curves for the citrus leaf δ^15^N values for the organic and conventional orchards showed that data from conventional orchards followed a normal distribution (Shapiro–Wilk normality test = 0.978, *P* = 0.956) and data from organic orchards followed a bimodal distribution (Fig. 5). Although there was a definite tendency for conventional citrus orchards to have lower δ^15^N values, there was overlap between the δ^15^N values for the organic and conventional citrus orchards (Fig. 5). Ninety-nine percent (*p* = 0.01) of the modeled conventional δ^15^N values would be expected to have a δ^15^N value below 10‰. This means that δ^15^N values above 10 ‰  could be expected to be obtained from organic citrus orchards, whereas δ^15^N values below 10 ‰ could not be unambiguously identified as organic or conventional.

## Discussion

Our data indicate significant differences in the citrus leaf δ^15^N values between organic and conventional citrus orchards. Despite this difference, given the high variability of the δ^15^N values, mostly in organic citrus orchards, it would be impractical to use a single analysis of the δ^15^N values in the citrus leaves to discriminate or confirm if an orchard has been following organic or conventional fertilization managements. However, the tendency for conventional citrus to have lower δ^15^N values allows us to define a limit value of 10 ‰ above which the young citrus leaves δ^15^N values obtained could be considered organic at 99% confidence level, whereas citrus leaves with δ^15^N values below 10‰ could not be confidently assigned to either fertilization treatment. The application of δ^15^N analysis to discriminate between organic and conventional cultivation has been discussed in detail in previous studies (Bateman, Kelly & Woolfe, 2007; Rogers, 2008). Here we present the first analysis of δ^15^N from young citrus leaves as an indicator of the orchard fertilization management. We found that the average δ^15^N values per orchard do not vary between seasons (Fig. 3), which makes the sampling season a non-critical factor for future studies. Our study also shows a high variability in the δ^15^N values from plant tissues sampled within citrus orchards with the same fertilization management, especially in organic orchards (Fig. 4). These differences in the 21 citrus orchards tested may be due to different reasons, all of them influenced directly by biochemical processes during fixation of N in the soil (Handley & Raven, 1992). The high variation in the δ^15^N values obtained within the organic treatments may be due to differences of treatments in their fertilization regimens (Table 1). Additionally, the irrigation frequency has an important effect on the decomposition of the organic matter and this may influence the N of organic origin available for the plant in the soil (Amundson et al., 2003). The citrus orchards used in this study belong to different growers that use different irrigation frequency and were located in areas with different soil types and structure. All these factors may add variability to δ^15^N values. Previous studies already showed a greater variability in the δ^15^N values in the organic groves (Bateman, Kelly & Woolfe, 2007). Remarkably, Legaz et al. (1982) found that N in citrus goes from old tissues to new tissues and is replaced, in those old tissues, by the N that comes from fertilization every growing season. Therefore, the δ^15^N measured in young leaves in this study comes mostly from the N reserve in old leaves which their N supply was replenished with N coming from the fertilizer applied the previous year, that means that is not replenished with the N from the last fertilization. Consequently, the time passed since each citrus orchard first adopted an organic fertilization management may be key to identify that orchard based on δ^15^N value. The average δ^15^N value obtained in this study for the conventional citrus orchards (∼5.2 ‰ for young leaves) was slightly higher than the average δ^15^N values obtained in previous studies in citrus with the same fertilization management (∼4.4‰ for fruit pulp) (Rapisarda et al., 2005). This slight increase may be due to the contribution of reactive nitrogen deposition in the soil from the traffic-derived NO_2_ (Ammann et al., 1999; Redling et al., 2013). It is widely known the high intensity traffic in the highways of Southern California, and it had been demonstrated that the plant material close to road traffic increase its δ^15^N by assimilating the nitrogen deposition from the traffic-derived NO_2_ (Ammann et al., 1999; Redling et al., 2013). The effect of this nitrogen deposition on the δ^15^N values is not adequately studied in commercial crops, even so this effect cannot explain the wide range in the δ^15^N values found in this and previous studies in organic crops (Bateman, Kelly & Woolfe, 2007; Rogers, 2008).

We found a significant correlation between the *D. citri* adults and host plant δ^15^N values in the field study on citrus trees and in the lab experiment on *M. koenigii* plants (Figs. 1 and 2). However, because of the high variability in the δ^15^N values between citrus orchards with the same fertilization management, especially in the organic ones, it has proved difficult to pinpoint *D. citri* nymphal natal origin using δ^15^N insect values. On average, the δ^15^N values for *D. citri* increased 0.4‰ for each unit increase in the values from the plant material where they were feeding on until they reached adult stage (young citrus leaves from the same exclusion cage). This result is consistent with the typical trend of δ^15^N values in animal tissues (second trophic level), which shows considerable enrichment compared to the δ^15^N values from the plants (first trophic level) (Rossmann, 2001).

In the laboratory experiments, we were able to establish the origin (organic or conventional treatment) of *D. citri* adults when the insects were feeding on plants with the same fertilization treatment throughout all nymphal stages. The δ^15^N values of *D. citri* adults that were transferred to the opposite fertilization treatment in the 2nd or 4th nymphal stage, had the δ^15^N values correlated with the δ^15^N values of leaves of the last fertilization treatment where they were feeding on (Fig. 1B). There were no significant differences in the δ^15^N values of *D. citri* adults that were switched from one fertilization treatment to another before they were three days old (Fig. 1B). Those *D. citri* adults that switched diet after ∼seven days old had δ^15^N values similar to those of the plants that they were feeding on as nymphs (Fig. 1B). In addition, the *D. citri* adults used as initial population (F_0_) in the assay “Impact of developmental stage on *Diaphorina citri* δ^15^ N incorporation” had no significantly different δ^15^N values after being fed on plant of different fertilization treatment for five days (oviposition period). All these results suggest that *D. citri* acquire the δ^15^N values mostly at the nymphal stage, and as newly emerged adults (less than three days old) (Fig. 1B). This may be due to the sclerotization process after adult emergence. The isotope label is fixed into structural body tissue of the organism at different moments depending if there are metabolically active or inert tissues (Rubenstein & Hobson, 2004). During the sclerotization process of the insect that happens after emergence, it may take high quantities of N from the plant and this N may fix in the exoskeleton of the insect, leaving the δ^15^N imprint of the last meal before sclerotization (Andersen, 2010).

## Conclusion

Stable isotopes have been proven to accurately reflect sources of nutrients in food webs and to unravel the migratory movements of insects. However, it does require a clear understanding of these isotopic patterns in nature and these studies should include the following steps. First, select a tissue that represents the appropriate temporal period of integration of geographical information, isotopically characterizing and differentiating among populations of interest, and finally linking populations by inferring geographical (or plant) origins based on isotopic similarity (Rubenstein & Hobson, 2004). Here, we selected a tissue, the young citrus leaves, which represents the appropriate temporal period and niche of *D. citri*. We tried to differentiate among organic vs. conventional fertilization managements and we linked the *D. citri* populations by inferring plant origin based on δ^15^N similarity (with positive results in the laboratory). However, we were not able to correlate all these data to pinpoint the origin of *D. citri* due to the enormous variability in the fertilization managements used in citrus orchards, especially in the organic fertilization management.

##  Supplemental Information

10.7717/peerj.8807/supp-1 Figure S1 Workflow chart of the experiment on the impact of developmental stage on *Diaphorina citri* *δ*^15^N values incorporation Draw credit: Francesc Gomez Marco.

10.7717/peerj.8807/supp-2 Data S1 N isotope values for all the experiments

## Additional Information and Declarations

### Field Study Permissions

The following information was supplied relating to field study approvals (i.e., approving body and any reference numbers): Field experiments were approved by the United States Department of Agriculture’s Animal and Plant Health Inspection Service (APHIS) (project number: #16-8130-0668-CA). Access to the sites was granted by the growers.

### Data Availability

The following information was supplied regarding data availability: The raw data is available in a Supplementary Files.
